# Critical evaluation of *Cbx7* downregulation in primary colon carcinomas and its clinical significance in Chinese patients

**DOI:** 10.1186/s12885-015-1172-6

**Published:** 2015-03-18

**Authors:** Xiang Zheng, Jing Zhou, Baozhen Zhang, Jun Zhang, James Wilson, Liankun Gu, Budong Zhu, Jin Gu, Jiafu Ji, Dajun Deng

**Affiliations:** 1 Key Laboratory of Carcinogenesis and Translational Research (Ministry of Education), Division of Cancer Etiology, Peking University Cancer Hospital & Institute, Beijing, China; 2 GRU Cancer Center, Georgia Regents University, Augusta, GA 30912 GA USA; 3 Department of Oncology, Peking University Cancer Hospital & Institute, Fu-Cheng-Lu #52, Beijing, 100142 China; 4 Department of Surgery, Peking University Cancer Hospital & Institute, Fu-Cheng-Lu #52, Beijing, 100142 China

**Keywords:** *Cbx7*, Quantitative RT-PCR, *Alu*, Colon carcinoma, Metastasis, Overall survival

## Abstract

**Background:**

CBX7 is a Polycomb group protein that shows variable expression changes in various cancers that are often contradictive. A mouse knockout experiment has validated the tumor suppressor role in carcinogenesis. The purpose of this study is to verify the tumor suppressor role of *Cbx7* in human colon carcinomas (CC).

**Methods:**

Frozen CC and the surgical margin (SM) tissue samples from patients (n = 97) were obtained from the Peking University Cancer Hospital. All patients had follow-up data for at least three years. The level of *Cbx7* mRNA and protein was determined by quantitative RT-PCR, immunohistochemistry and Western blot, respectively. The association between *Cbx7* mRNA level and clinicopathological characteristics of CC patients was then statistically analyzed.

**Results:**

CBX7 expression changes detected through immunohistochemistry and Western blot in 10 pairs of representative CC samples significantly correlated with their corresponding mRNA levels when *Alu*, but not *GAPDH*, was used as the endogenous reference control in quantitative RT-PCR. The *Alu*-normalized *Cbx7* mRNA levels were significantly increased in SM tissues when compared with CC tissues or colon biopsies taken from non-cancer patients (Student’s *t*-test, *P* < 0.036 or 0.007). Furthermore, decreased levels of *Cbx7* mRNA positively correlated with lymph metastasis (*P* = 0.029). Overall survival (OS) of CC patients classified as *Cbx7* expression-low was considerably shorter than those classified as *Cbx7* expression-high (Hazard ratio = 2.97, 95% CI [1.68 ~ 5.25]; *P* <0.001). Multiple variant analyses showed that the *Cbx7* expression-low was an independent predictor of short OS (Hazard ratio = 3.16, 95% CI [1.58-6.30]; *P* < 0.001).

**Conclusion:**

*Cbx7* is downregulated in CCs, and *Cbx7* expression-low tumors correlated with lymph metastasis and poor overall survival of CC patients.

**Electronic supplementary material:**

The online version of this article (doi:10.1186/s12885-015-1172-6) contains supplementary material, which is available to authorized users.

## Background

The Polycomb-group (PcG) proteins function as epigenetic transcriptional regulators through multiple mechanisms. PcG proteins are mainly categorized into two groups, polycomb repressive complex 1 (PRC1) and 2 (PRC2) [1]. CBX7 is a Polycomb protein that has shown tumor suppressive function and is a component of PRC1. Recent studies have utilized *cbx7-*knockout mice to validate the tumor suppressor role of *cbx7* in liver and lung carcinogenesis [2]. However, analysis of many types of cancers revealed that the *Cbx7* expression levels were significantly altered, but the changes were often considerably contradictive. While some studies showed that *Cbx7* behaved as an oncogene in lymphoma, prostate cancer, ovarian cancer, and gastric cancer [3-6], other studies indicated *Cbx7* was acting as a tumor suppressor gene in the bladder, colon, pancreas, and thyroid cancers [7-11]. Currently, the reasons leading to these conflicting results have not been investigated.

Colon carcinoma (CC) is the third most common cancer and the fourth leading cause of cancer death worldwide [12]. One large-scale patient study indicated that the loss of *Cbx7* expression in CC correlates with poor prognosis and short survival using results analyzed through immunohistochemistry in tissue microarray [11]. Using quantitative RT-PCR (qRT-PCR), similar results were obtained among 22 Chinese CC patients [13]. The qRT-PCR assay offers significant advantages over the hemi-quantitative immunohistochemistry assays for detecting *Cbx7* expression changes in cancers. In the present study, a qRT-PCR assay capable of correlating *Cbx7* mRNA level with protein expression was developed, and the *Cbx7* transcription levels from 97 CC patients were quantified and statistically analyzed for clinicopathological correlations.

## Methods

### Clinical samples

CC and surgical margin (SM) tissue specimens were obtained from surgical resection patients without neo-adjuvant therapy (n = 97) in Peking University Cancer Hospital (Beijing, China) from 2004 to 2011. The patient population contained 49 males and 48 females with a mean age of 62 years (range 34–89), who were not included in the previous study [13]. Tumors were staged using the tumor-node-metastasis (pTNM) staging of the International Union Against Cancer (2003) [14]. The number of patients staged from I to IV were 1, 33, 36 and 27, respectively (Table 1). The SM tissues were more than 5 cm from the tumor and were validated by an experienced pathologist. None of the patients received preoperative chemotherapy. All patients had follow-up data for at least 36 months (*median* 61). 49 CC patients were post-operatively treated with adjuvant chemotherapy (Folfox for 30 patient). 45 patients (46.4%) suffered recurrent CC and 52 patients (53.6%) died during follow-up. Paraffin blocks were selected from suitable formalin-fixed paraffin-embedded tissue with an average age of 62.2 years (range 34 ~ 89). Normal colon biopsies were obtained from non-cancerous patients (n = 51). The Institutional Review Boards of Peking University Cancer Hospital and Institute approved the study. All samples were obtained with the patients’ informed written consent.

**Table 1:** **Association of** ***Cbx7*** **mRNA level and clinicopathological features of colon cancer patients**

Clinicopathological features		*n*	*Cbx7*mRNA level^a^	
			Colon cancer	Surgical margin
Age (years)	≤62	42	**2.69 (1.07-4.61)** ^**b**^	2.42 (1.31-4.88)
	>62	55	**1.38 (0.44-3.37)**	1.72 (0.98-4.43)
Sex	Male	49	2.04 (0.74-4.54)	1.77 (0.97-4.00)
	Female	48	1.60 (0.90-3.40)	2.22 (1.31-5.29)
Location	Ascending/Transverse	42	1.94 (1.05-4.52)	1.70 (1.27-3.27)
	Descending/Sigmoid	37	1.90 (0.72-4.61)	1.94 (1.04-6.26)
	Undefined	18	1.29 (0.61-2.75)	2.42 (1.20-7.74)
Differentiation	Poor	24	1.60 (0.89-3.59)	1.60 (1.31-3.54)
	Moderate/Well	70	2.02 (0.76-4.59)	2.22 (0.949-6.05)
Vascular invasion	Absent	69	1.95 (0.79-4.48)	1.94 (1.27-5.25)
	Present	26	1.74 (0.69-4.13)	1.90 (1.13-4.71)
pTNM	I&II	39	2.06 (1.01-4.54)	1.94 (0.66-5.41)
	III&IV	58	1.63 (0.69-3.92)	1.90 (1.30-4.66)
Invasion	T1 ~ 2	5	5.77 (4.48-8.25)	1.31 (0.30-3.09)
	T3	51	1.40 (0.38-3.73)	2.39 (0.94-5.83)
	T4	41	1.83 (0.96-3.81)	1.82 (1.31-3.39)
Lymph metastasis	Negative	50	**2.54 (1.07-4.57** **)** ^**b**^	1.77 (1.18-4.63)
	Positive	47	**1.29 (0.24-2.94)**	2.22 (1.12-4.85)
Distant metastasis	Negative	69	2.04 (0.96-4.27)	1.94 (1.08-4.95)
	Positive	29	1.31 (0.42-4.56)	1.97 (1.22-5.15)
Post-operative therapy	No	48	1.93 (0.75-3.77)	1.75 (1.03-5.03)
	Yes	49	1.64 (0.94-4.48)	2.22 (1.27-4.00)

^a^*Alu*-normalized relative copy number (×10^−4^), the value is presented *median* (25% ~ 75% percentile); ^b^Mann–Whitney test, *P* = 0.007

### qRT-PCR

Total RNA was extracted from 30–50 mg of tumor tissue using a commercial RNA isolation kit according to the manufacturer’s protocol (Ultrapure RNA Kit, CWBIO, Beijing). Subsequently, the RNA concentration was checked using 1.0% agarose gel electrophoresis stained with 0.5 μg/ml ethidium bromide and quantified with a NanoVue spectrophotometer (GE Healthcare). For reverse transcription, 1 μg RNA, 20 units reverse transcriptase, 1× reaction buffer, 1 mM deoxynucleotides, 3 mM MgCl_2_, and 4.0 mg of random hexamers were used. The reaction mixtures were incubated at 25°C for 10 min, 42°C for 1 h, and 95°C for 5 min according to the manufacturer’s protocol (Improm-II Reverse Transcription System A3800, Promega, USA). The cDNA was stored at −20°C.

qRT-PCR was performed using an ABI 7500 Fast Realtime System (Applied Biosystems, Foster City, CA, USA). Primers and a TaqMan probe for *Cbx7* were designed and synthesized according to the Taqman Gene Expression Assay (Roche Diagnostics, Mannheim, German). The primer sequences follow: human *Cbx7* gene 5′-cgtcatggcctacgagga-3′ (sense), 5′-tgggtttcggacctctctt-3′ (antisense); TaqMan probe 5′-FAM-aggaggag-TEMER-3′ [8,15]; *GAPDH* 5′-gaaggtgaaggtcggagt-3′ (sense) and 5′-gaagatggtgatgggatttc-3′ (antisense); the *Alu* elements 5′-gaggctgaggcaggagaatcg-3′ (sense), 5′- gtcgcccaggctggagtg-3′ (antisense) [16]. PCR reactions were carried out in a final volume of 10 μL containing 5 μL Maxima Probe/ROX qPCR Master Mix (2×) (K0233, Thermo Scientific), 0.5 μM of each primer and DNase-free water. The PCR conditions were 5 min at 95°C, followed by 40 cycles of 95°C for 15 s, 54°C for 30s, and 72°C for 35 s and finished with a melting curve analysis. The relative copy number [2^-ΔCT^] of *Cbx7* mRNA was determined from the difference in cycle threshold (CT) values between the target and reference genes.

### Immunohistochemistry (IHC)

The paraffin was removed from the embedded CC and SM tissue samples using xylene. The samples were then rehydrated in a graded series of ethanol solutions. Antigen retrieval was performed in Tris/EDTA (pH 9.0) for 3 min at 120°C. The sections were then incubated for 20 min in 3% H_2_O_2_ and washed with 0.025% Triton X-100/TBS (TBST). Blockage was performed with 10% goat serum for 2 h at room temperature. The slides were then incubated overnight at 4°C with anti-CBX7 monoclonal antibody (ab21873, Abcam, Cambridge, UK). Subsequently, the sections were incubated with an HRP-conjugated anti-mouse EnVision system (DAKO, Glostrup, Denmark) for 20 min at 37°C followed by staining with diaminobenzidine hydrochloride (DAB, DAKO). Normal mouse IgG was applied as negative control (Additional file 1: Figure S1). The sections were counterstained with hematoxylin. The intensity of nuclear CBX7-staining in the epithelial and stromal cells was grouped as negative (−), weak (visible at high magnification = +1), moderate (visible at low magnification = +2), or strong (strikingly positive at low magnification = +3).

### Western blot

Whole-cell protein extracts were prepared from primary tumors. The samples underwent electrophoresis in a 12% SDS-PAGE gel followed by blotting onto a Polyvinylidene-Fluoride membrane (Bio-Rad). The membranes were incubated in PBS containing 5% skim milk and 0.05% Tween-20 for 2 h at room temperature, then probed overnight with anti-CBX7 antibody (1:1000) (Abcam) in blocking solution at 4°C. An HRP-labeled goat anti-rabbit secondary antibody was then used (DAKO K5007, Glostrup, Denmark). The results were visualized on a Fluor chem system (Cell Biosciences). Integrated Option Density (IOD) was used to quantify the amounts of CBX7 and β-Actin. β-Actin was used as a reference control. In order to normalize the levels of CBX7 between samples, the IOD_CBX7_/IOD_Reference_ ratio was calculated.

### *Cbx7* plasmid construction and transfection

The coding region of *Cbx7* was inserted into the pEGFP-C1 vector and used to transfect cultured cells as described previously [15].

### Transwell migration and matrigel invasion assays

The migration and invasion capacity of colon cancer cell lines SW480 (5 × 10^4^ cells/well) and HCT116 (4 × 10^4^ cells/well) (kindly provided by Dr. Yuanjia Chen at Peking Union Medical College Hospital) were tested using the Transwell migration and invasion assays 48 hrs after transiently transfection with the *Cbx7* plasmid or empty vectors for 48 hrs. [15].

### Formation of pulmonary tumor in nude mice

SW480 cells (2 × 10^6^ cells in 0.2 ml) were injected into SCID mice via the tail vein 48 hrs after transient transfection with the *Cbx7* plasmid or empty vectors. The mice (8/Group) were harvested during the 6^th^ experimental week. Chest wall, number of pulmonary metastasis tumor nodules, and the lung weight were then measured for each mouse. The lung organs were fixed with Bouin solution, paraffin-embedded and cut into 5 μm slides along the maximum area, and examined microscopically following H.E. staining.

### Statistical analysis

All statistical analyses were performed using SPSS software (SPSS version 17.0). The correlation between CBX7 protein level and mRNA level was analyzed using the Spearman’s rank correlation coefficient or the Pearson product–moment correlation coefficient. The nonparametric Wilcoxon test, Kruskal-Wallis test, and Mann–Whitney test were applied to evaluate the association between *Cbx7* transcription and clinicopathological tumor features. Kaplan–Meier survival curves were generated and compared using the log-rank test. A multivariable Cox regression model was applied to determine if a particular factor was an independent predictor of survival in multivariate analysis. All statistical tests were two-sided, and *P*-values < 0.05 were considered statistically significant.

## Results

### qRT-PCR optimization for quantifying *Cbx7* mRNA levels in colon tissues

**Figure 1:**
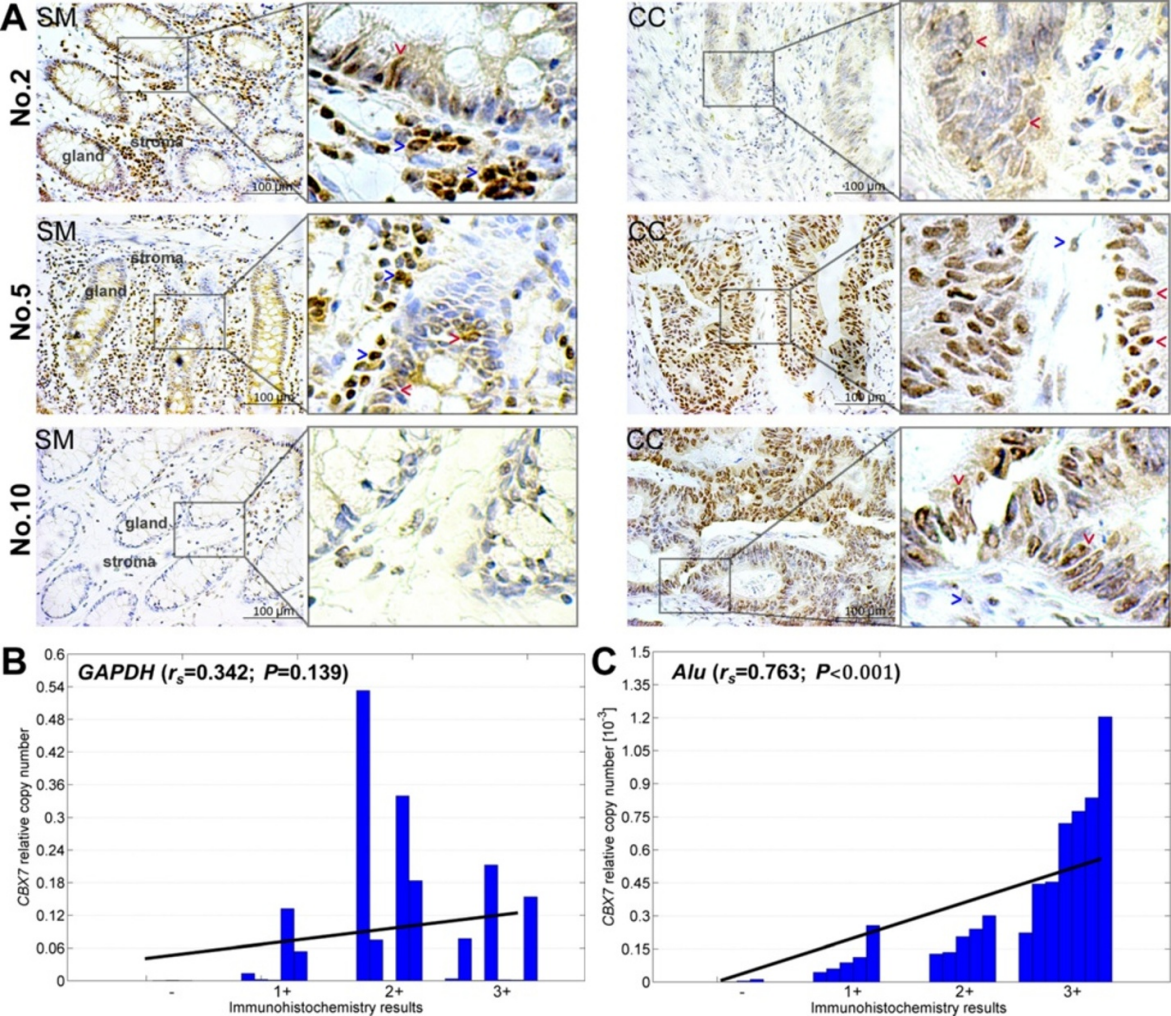
**Correlation analysis between CBX7 expression in immunohistochemical (IHC) analysis and quantitative RT-PCR using different reference genes. (A)** CBX7-IHC images for CC and SM samples from three patients; Strong nuclear CBX7 protein staining was mainly located in cancer cells (red-arrows) in the representative colon cancer (CC) tissues, but located in both glandular epithelial cells and lymphoid cells (blue-arrows) in the corresponding surgical margin (SM). **(B)** CBX7-IHC staining scores and *GAPDH*-normalized *Cbx7* mRNA levels; **(C)** CBX7-IHC staining scores and *Alu*-normalized *Cbx7* mRNA levels. The relative copy number of *Cbx7* mRNA was determined from the difference in cycle threshold values between the target and reference genes.

*GAPDH* mRNA is traditionally used as the reference control in qRT-PCR; however, the transcription of *Alu* elements is a novel reference control that has recently been developed to replace *GAPDH* [16]. In order to identify the optimal reference control for our study, qRT-PCR was run using both the *Alu* and *GAPDH* reference controls to determine the correlation between CBX7 protein levels (used as the golden standard) and *Cbx7* mRNA levels in the same set of human colon tissues. The amount of CBX7 protein in the paired CC and SM samples (n = 10) was analyzed using the IHC assay. Results of IHC analysis revealed diverse patterns of CBX7 expression changes in CC relative to the corresponding SM samples. While CBX7 expression was decreased in some CC samples (Figure 1 No.2), it was increased (Figure 1 No.10) or unchanged in other samples (Figure 1 No.5). In addition, the CC tissues revealed strong CBX7 protein expression in the nucleus of cancer cells. However, nuclear CBX7 staining was more prevalent in the glandular epithelial cells and stromal lymphoid cells in the corresponding SMs (Figure 1) as well as normal colon biopsies from non-cancer patient controls (Additional file 1: Figure S1).

Consistent with our previous study [13], the amount of *Cbx7* mRNA in the 10 pairs of CC tissues was significantly lower than the SM tissues when *GAPDH* was used as the reference in qRT-PCR analysis (*P* = 0.013); however, the *GAPDH*-normalized *Cbx7* mRNA level was not correlated with the amount of CBX7 protein detected in the IHC assay (Spearman’s rank correlation coefficient, *r*_s_ = +0.204, *P* = 0.460; Figure 1B). In contrast, when *Alu* was used as the reference control the CC and SM samples did not show a significant difference in *Cbx7* mRNA levels (*P* = 0.939); however, the *Cbx7* mRNA levels strongly correlated with the amount of CBX7 protein (Spearman's rank correlation coefficient, *r*_s_ = +0.763, *P* < 0.001; Figure 1C).

**Figure 2:**
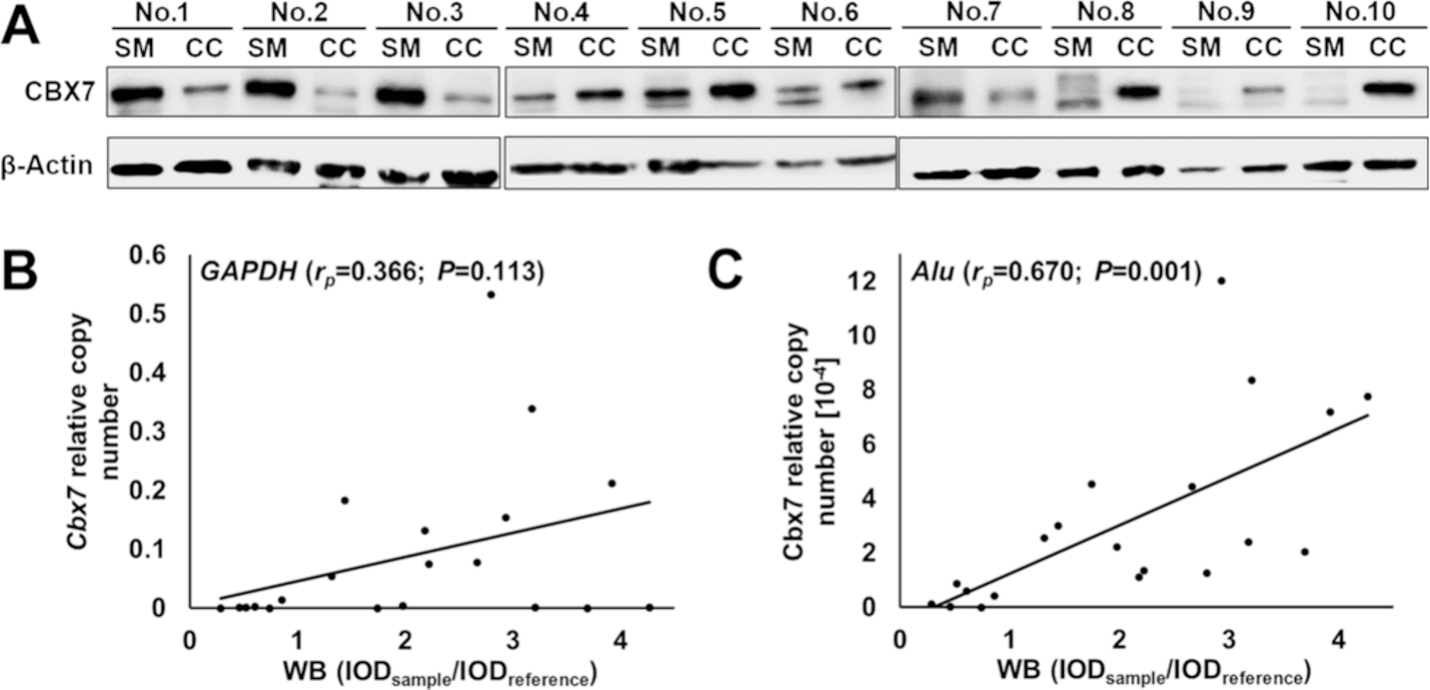
**Correlation analysis between CBX7 expression in Western blot analysis and quantitative RT-PCR using different reference genes. (A)** Detection of CBX7 protein in CC and SM samples using Western blot analysis; **(B)** Relative intensities of CBX7 protein to β-Actin and *GAPDH*-normalized *Cbx7* mRNA levels; **(C)** Relative intensities of CBX7 protein to β-Actin and *Alu*-normalized *Cbx7* mRNA levels.

In order to verify the IHC results, Western blot was performed on the CC and SM samples to validate the CBX7 protein levels. The results were consistent with IHC (Figure 2A). The positive correlation between CBX7 protein level and mRNA level in the *Alu* control samples was confirmed (Pearson product–moment correlation coefficient, *r*_p_ = +0.670, *P* = 0.001), and no correlation was seen in the *GAPDH* control samples (*r*_p_ = +0.366, *P* = 0.113) (Figure 2B, C). Therefore, the *Alu* transcript was used for qRT-PCR analysis.

**Figure 3:**
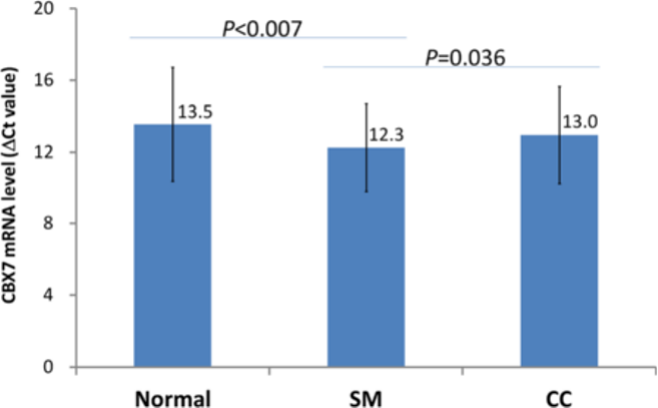
**Comparison of*Cbx7*mRNA levels among Normal, CC and SM samples.** The mRNA level represented as the ∆CT value between *Alu* and *Cbx7* transcripts. A higher ∆CT value indicates a lower mRNA level.

In order to investigate whether there was a significant difference in *Cbx7* transcription between CC and SM tissues, a larger sample size of patients (n = 97) was analyzed. Results showed that the average level of *Cbx7* mRNA was significantly lower in CC tissues than SM tissues (∆CT value: 13.0 vs. 12.3; Student’s *t*-test, *P* = 0.036; Figure 3). However, the *Cbx7* mRNA level in SM tissue was also significantly higher than normal colon tissue controls (n = 51) (∆CT value: 12.3 vs. 13.5; *P* < 0.007, Figure 3). Taken together, the relative copy numbers of *Cbx7* mRNA in the SMs and CCs are 245% and 151% of that in the normal controls, respectively. These results suggest that *Cbx7* transcription is considerably upregulated in the SMs.

### Downregulation of *Cbx7* expression correlated with poor prognosis of CC patients

Association analysis showed that CC patients with lymph metastasis had lower *Cbx7* mRNA levels than their negative counterparts (Table 1; *P* = 0.029). Furthermore, younger patients (cutoff age, 62 years old) displayed significantly higher *Cbx7* mRNA expression levels than older patients (*P* = 0.027). Significant associations were not observed between patients with different gender, tumor differentiation, vascular invasion states, invasion, distant metastasis, or pTNM stage. In addition, the *Cbx7* mRNA levels in the SM samples were not associated with clinicopathological features.

**Figure 4:**
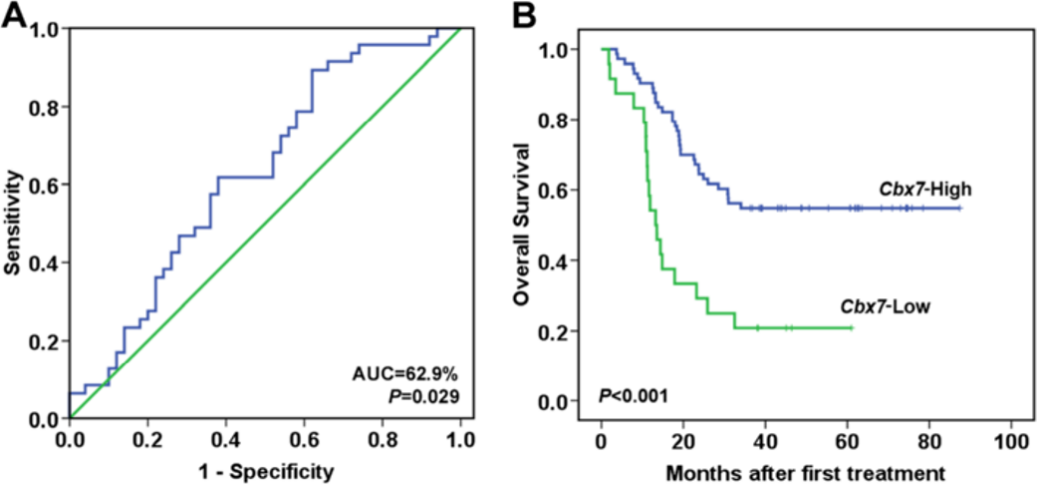
***Cbx7*mRNA level in CCs correlates with prognosis. (A)** ROC curve of CC metastasis used to classify patients as *Cbx7-high* or *low* (cut-off value = 7.45 × 10^−5^); the area under the curve (AUC) is 62.9% (*P* < 0.029); **(B)** Kaplan-Meier overall survival curves for patients classified as *Cbx7* expression-low and –high (*P* = 0.001, log-rank test).

Using the *Cbx7* mRNA level in CC tissues to detect metastasis, the integral (AUC) of the receiver operating characteristic (ROC) curve was 62.9% (Figure 4A; *P* = 0.029). By using the relative copy number of 7.45 × 10^−5^ as the cut-off value, patients classified as *Cbx7* mRNA-low had a shorter overall survival (OS) than patients classified as *Cbx7* mRNA-high (hazard ratio = 2.97; 95% CI [1.68 ~ 5.25]; *P* < 0.001) (Figure 4B). The three-year survival rates were 20.8% (5/24) and 54.8% (40/73) for the *Cbx7* mRNA-low and -high patients, respectively. Multivariable analysis revealed that *Cbx7* mRNA level was an independent factor for OS after adjusting for vascular invasion, pTNM stage, age, sex, and differentiation (hazard ratio = 3.16, 95% CI [1.58-6.30], *P* <0.001; Table 2).

**Table 2:** **Multivariate analysis of overall survival in colon cancer patients**

Variable	Hazard ratio (95% CI)	*P*-value
*Cbx7* mRNA-low	3.16 (1.58-6.30)	0.001
Vascular invasion	3.43 (1.64-7.17)	0.001
pTNM	4.13 (1.71-9.98)	0.002
Age	1.31 (0.65-2.64)	0.449
Sex	0.70 (0.38-1.29)	0.255
Differentiation	0.79 (0.39-1.61)	0.512
Adjuvant therapy	1.05 (0.55-2.01)	0.888

### Effects of enforced *Cbx7* overexpression on migration and invasion of cancer cells

**Figure 5:**
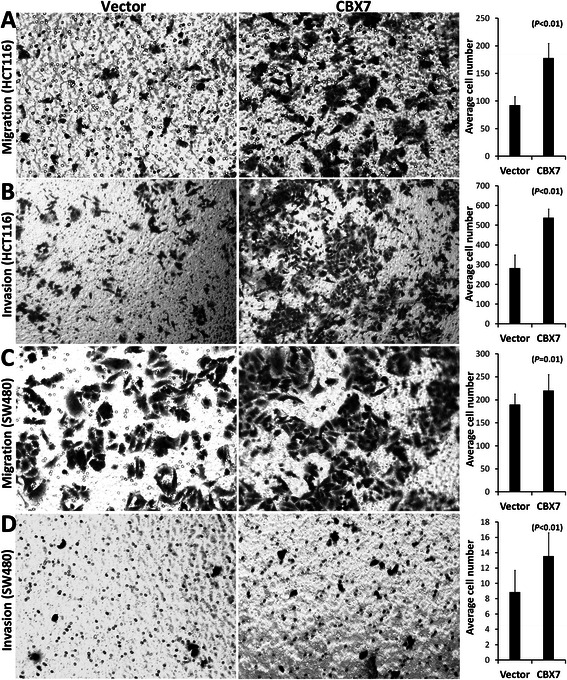
**Enforced*Cbx7*overexpression promotes migration of colon cancer cell lines. (A and B)** Results of Transwell migration and Matrigel invasion tests using HCT116 cells transiently transfected with the *Cbx7* expression vector for 48 hrs, respectively; **(C and D)** Results of Transwell migration and Matrigel invasion tests using SW480 cells transiently transfected with the *Cbx7* expression vector for 48 hrs, respectively. The transfection efficiency is presented in Figure S2.

The Transwell migration and Matrigel invasion assays were further used to study whether CBX7 directly affects metastasis of cancer cells transient transfected with the *Cbx7* expression vector (Additional file 2: Figure S2). Results showed that transient *Cbx7* overexpression significantly increased migration and invasion of colon cancer HCT116 cells (Figure 5A, B). Similar results were also observed in another colon cancer cell line SW480 (Figure 5C, D). Proliferation of these cells was not affected by *Cbx7* overexpression (data not shown).

**Figure 6:**
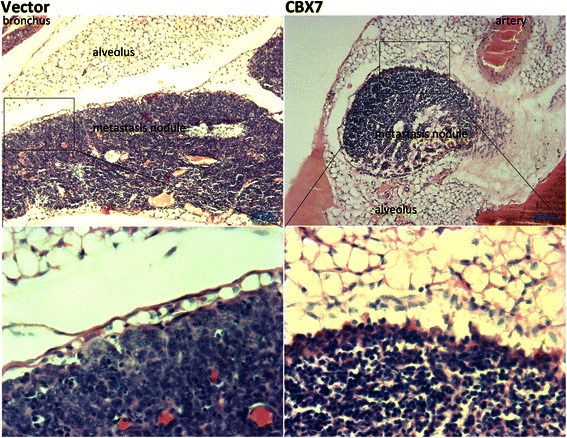
**Images of experimental pulmonary metastasis nodules of colon cancer SW480 cells with or without*Cbx7*overexpression in SCID mice**. Slides are prepared along the maximum lung area for each mouse.

In order to further elucidate the effect of CBX7 on cancer cell migration, SW480 cells transiently transfected with the *Cbx7* or control vector were injected into the tail vein of SCID mice, and the pulmonary metastases were subsequently analyzed (Figure 6). Six weeks after injection, the average lung weight of the mice in the CBX7 group (n = 8; 0.395 g ± 0.022) was similar with that in the vector control group (n = 8; 0.387 g ± 0.023). The positive rate of pulmonary metastasis in the CBX7 group (3/8) was slightly increased compared to the control group (1/8), but not statistically significant (Fisher-exact test, *P* = 0.57). Additionally, 3 pulmonary nodules were identified in the CBX7 group, compared to 2 in the control group. However, this difference was not significant.

## Discussion

The *Cbx7* expression levels in human cancers are highly variable and often contradictive. The organ/tissue-specificity, assay used for analysis, and sample differences may account for these conflicting results. In the present study, it was determined that by using *Alu* as the normalization control, *Cbx7* mRNA levels in the CC and SM tissues were positively and significantly correlated with the amount of CBX7 protein detected by both IHC and Western blot. Such a correlation was not observed when *GAPDH* was used as the normalization control. By using the *Alu* RNA reference, it was found that *Cbx7* transcription was significantly downregulated in CC and *Cbx7* expression-low tumors contributed to a high risk of lymph metastasis and poor overall survival in Chinese CC patients. These results are consistent with a previous report, which showed that loss of CBX7 expression in CC correlates with poor outcomes based on large-scale patient analysis by IHC in tissue microarray [11].

*GAPDH* is the traditional reference used in qRT-PCR to normalize mRNA levels in cell/ tissue samples containing different cell numbers. It is especially effective for samples of homogeneous cellularity/constitution; however, its reliability for normalization in samples that are cellularly heterogenic is problematic. This shortcoming is especially highlighted when attempting to compare gene mRNA levels in cancer tissues with corresponding normal tissues. A number of strategies have been proposed for proper data normalization in qRT-PCR [16-18]. There are about 800,000 copies of *Alu* elements in each haploid of the human genome. More than one million copies of the *Alu* element (about 300 bp) are located in untranslated regions (UTRs) of 1,500 genes. Therefore, transcriptional dysregulation of a few *Alu* elements has no significant affect on altering the total amount of *Alu* transcripts in the whole human transcriptome. This unique characteristic makes *Alu* transcripts a good reference gene for qRT-PCR. It is known that *Alu* elements are globally hypomethylated in cancers [19]; however, it is not known if the hypomethylation leads to upregulation of *Alu* expression in the genome and subsequently decreases its reliability. The high consistency between CBX7 protein level and *Alu*-normalized *Cbx7* mRNA level in colon tissues found in the present study is in strong agreement with recent reports suggesting that *Alu* RNA is a reliable reference in qRT-PCR analysis [16], and could be particularly beneficial for the evaluation of gene expression changes between cancer and normal tissues.

It has been reported that *Cbx7* transcription was downregulated among 22 Chinese CC patients using the typical *GAPDH*-normalized qRT-PCR analysis [13]. Although a similar result was also observed among the 10 pairs of CC and SM samples in the present study, the *GAPDH*-normalized *Cbx7* mRNA levels did not correlate with the amount of CBX7 protein detected through IHC and Western blot analysis. This implies that the observed difference in *GAPDH*-normalized *Cbx7* mRNA levels between CC and SM samples may not be an accurate indicator of protein level. Although other factors, such as stability and degradation differences, could have played a role in the inconsistency observed between mRNA and protein levels of a gene in tissues of interest, the high consistency and repeatability seen using the *Alu*-normalized *Cbx7* mRNA and CBX7 protein levels shows that selecting a suitable reference is very critical in drawing a reliable conclusion. Use of an unreliable reference gene for qRT-PCR analysis may contribute more to the noted inconsistency than has previously been estimated. It is increasingly necessary to determine if the correct normalization control is being used when qRT-PCR assays are utilized in studies on tumor biology [20,21].

Interestingly, although the average *Cbx7* mRNA level in SMs was significantly higher than CCs, it was also significantly higher than in normal colon control tissues from non-cancer patients. Age also seems to be associated with the levels of CBX7. The average level of *Cbx7* mRNA in CC and SM samples from patients ≤62 yrs was significantly higher than was observed in older patients. Therefore, because the CC patients were much older than the controls, the *Cbx7* mRNA level in the normal colon tissues distant from the site of malignancy (not available in the present study) should be lower than that from the non-cancer controls. In other words, the expected *Cbx7* expression difference between the SMs and distant normal colon from the CC patients might be higher than the observed difference between the SMs and normal colon biopsies from non-cancer patients. This suggests that alteration of *Cbx7* expression in colon carcinogenesis may be far more complex than expected.

According to the map of human proteome [22], CBX7 is highly expressed in B cells (Additional file 3: Figure S3). In the present study, strong nuclear CBX7 staining was frequently observed in the lymphoid cells in the SMs and the normal colon biopsies, but not in CC samples. It is likely that both the decreased number of CBX7-positive lymphoid cells in the CC tissues, and the increased number of lymphocytes infiltrating into stromal tissues around cancer cells contributes to the downregulation of *Cbx7* expression in CCs. It cannot be excluded that *Cbx7* may be upregulated in SMs as a B-cell infiltration-related host response to the presence of cancer cells. More studies are necessary to further elucidate the role of *Cbx7* in tumor development and modulation.

Contribution of CBX7 to cancer development may be organ-dependent [3-11]. CBX7 is suggested to help suppress the progression of human colon cancers [10,13]. The results of our study were in close agreement with this conclusion. However, the results of the Transwell tests and mouse experimental pulmonary metastasis assay showed that enforced *Cbx7* overexpression might slightly increase the migration/invasion capacity of colon cancer cells. This suggests that the role of the exogenous *Cbx7* overexpression in epithelial cancer cells might be different from that of the endogenous *Cbx7* in colon cancer. Whether downregulation of *Cbx7* in stromal lymphoid cells involves in the progression of colon cancers should be studied further.

## Conclusions

In conclusion, this study revealed that the total amount of *Alu* RNA is a suitable and accurate endogenous reference for qRT-PCR analysis. Most importantly, it was shown that *Cbx7* is downregulated in CC and *Cbx7* expression-low is associated with metastasis and short overall survival of CC patients.

## Additional files

Additional file 1: Figure S1. IHC analysis of CBX7 expression in normal colon biopsies from non-cancer patients. Strong nuclear CBX7 protein staining was located in both glandular epithelial cells (red arrows) and lymphoid cells (blue arrows) in the normal colon tissues. Bar, 100 μm.
Additional file 2: Figure S2. Transfection efficiency of CBX7 EGFP-C1 vector in cancer cell lines HCT116 and SW480. Both cytoplasm and nucleus EGFP proteins are observed in most cells at 48 hrs following transient transfection with the EGFP control vector (GFP-Ctrl). EGFP-CBX7 fusion proteins mainly locate in the nucleus of cells at 48 hrs following transfection with the EGFP-CBX7 vector (GFP-CBX7).
Additional file 3: Figure S3. CBX7 protein level in various human tissues and cells analyzed using high-resolution Fourier-transform mass spectrometry. This map has been downloaded from the Human Proteome Map Web site (http://www.humanproteomemap.org) (Ref: [22]).

## Abbreviations

CC: Colon carcinomas
SM: Surgical margin
PcG: Polycomb-group
PRC: Polycomb repressive complex
qRT-PC: Quantitative RT-PCR
pTNM: Tumor-node-metastasis
CT: Cycle threshold
IHC: Immunohistochemistry
WB: Western blot
IOD: Integrated option density
