# Supplementary figures and images for: Critical evaluation of Cbx7 downregulation in primary colon carcinomas and its clinical significance in Chinese patients
Source: BMC Cancer. 2015 Mar 18;15:145. doi: 10.1186/s12885-015-1172-6 (PMC4365772; doi:10.1186/s12885-015-1172-6)

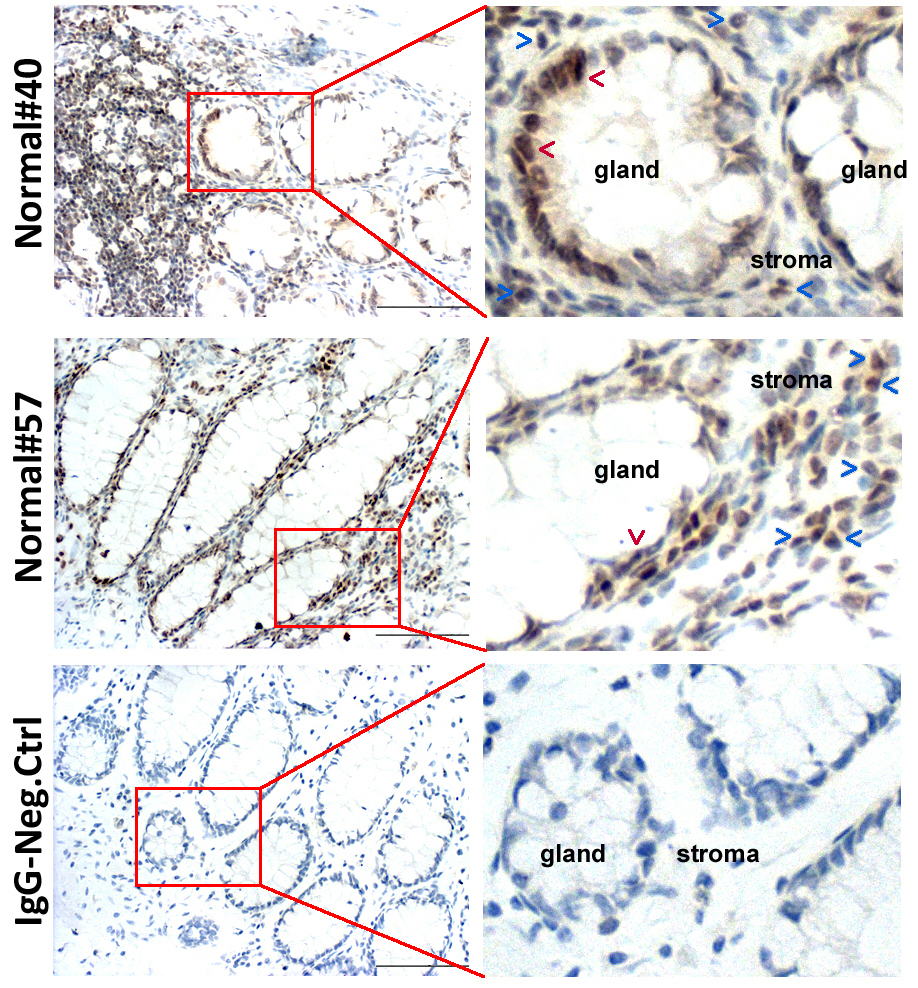

Supplement: Additional file 1: Figure S1. — IHC analysis of CBX7 expression in normal colon biopsies from non-cancer patients. Strong nuclear CBX7 protein staining was located in both glandular epithelial cells (red arrows) and lymphoid cells (blue arrows) in the normal colon tissues. Bar, 100 μm. [file 12885_2015_1172_MOESM1_ESM.jpeg]

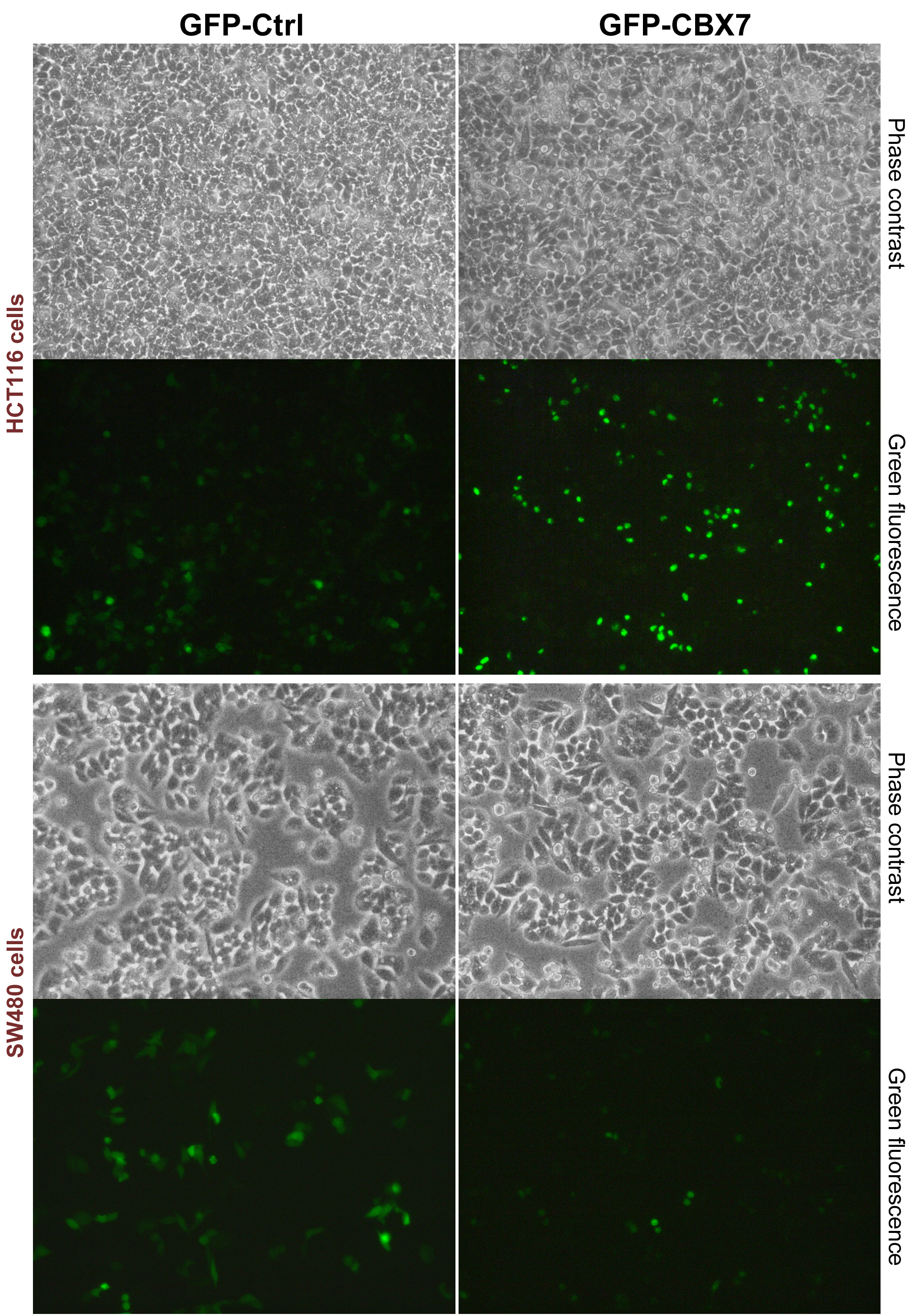

Supplement: Additional file 2: Figure S2. — Transfection efficiency of CBX7 EGFP-C1 vector in cancer cell lines HCT116 and SW480. Both cytoplasm and nucleus EGFP proteins are observed in most cells at 48 hrs following transient transfection with the EGFP control vector (GFP-Ctrl). EGFP-CBX7 fusion proteins mainly locate in the nucleus of cells at 48 hrs following transfection with the EGFP-CBX7 vector (GFP-CBX7). [file 12885_2015_1172_MOESM2_ESM.jpeg]

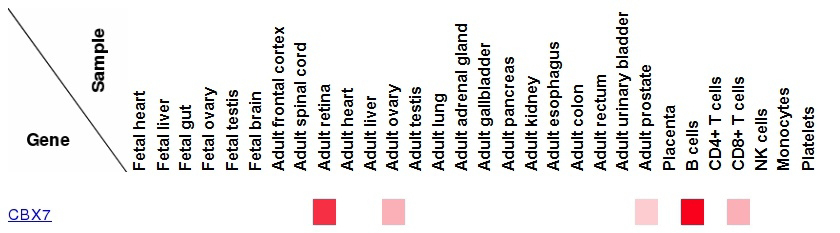

Supplement: Additional file 3: Figure S3. — CBX7 protein level in various human tissues and cells analyzed using high-resolution Fourier-transform mass spectrometry. This map has been downloaded from the Human Proteome Map Web site (http://www.humanproteomemap.org) (Ref: [22]). [file 12885_2015_1172_MOESM3_ESM.jpeg]
